# Supplementary material for: Impact of white blood cell count on the development of contrast-induced acute kidney injury in patients receiving percutaneous coronary intervention
Source: PeerJ. 2024 Jun 28;12:e17493. doi: 10.7717/peerj.17493 (PMC11636986; doi:10.7717/peerj.17493)
Supplement: Supplemental Information 2 [file peerj-12-17493-s002.docx]

STROBE Statement—checklist of items that should be included in reports of observational studies

|  | Item No. | Recommendation | Page  No. | Relevant text from manuscript |
| --- | --- | --- | --- | --- |
| **Title and abstract** | 1 | (*a*) Indicate the study’s design with a commonly used term in the title or the abstract | 1 | Impact of white blood cell count on the development of Contrast-Induced Acute Kidney Injury in patients receiving percutaneous coronary intervention |
|  |  | (*b*) Provide in the abstract an informative and balanced summary of what was done and what was found | 2 | This study aimed to investigate the efficacy of a pre-procedural white blood cell (WBC) count in the prediction of contrast-induced acute kidney injury (CI-AKI) risk in coronary artery disease patients receiving a percutaneous coronary intervention (PCI). This observational study comprises a sample of 1013 coronary artery disease patients (including ACS and stable angina) receiving PCI, gathered from September 2015 to July 2017.CI-AKI incidence in the study population was 4.8% (49/1013). Patients in the CI-AKI group had significantly higher WBC counts than those in the non-CI-AKI group (10.41± 5.37 VS 8.09 ± 3.10, P=0.004). Logistic analysis showed that WBC count (odds ratio [OR]: 1.12, 95% CI:1.03-1.21, P=0.006) was a significant and independent predictor of CI-AKI risk in patients receiving PCI, Receiver-operating characteristic (ROC) curve analysis found that pre-procedural WBC count ≥11.03*109/L was the optimal cut-off value in the prediction of CI-AKI risk with a sensitivity of 41.0% and a specificity of 86.5%. Patients with CI-AKI had a significantly worse 1-year survival rate than patients without CI-AKI (91.8% vs 97.6%, P =0.012). In summary, increased pre-procedural WBC count is associated with an increased risk of developing CI-AKI in patients receiving PCI. |
| Introduction | | | |  |
| Background/rationale | 2 | Explain the scientific background and rationale for the investigation being reported | 2-3 | Contrast-induced acute kidney injury (CI-AKI) is a common complication arising among patients receiving percutaneous coronary intervention (PCI) . The incidence of CI-AKI is 2% for the general population; however, patients receiving PCI are at higher risk of developing CI-AKI, and patients who also suffer from chronic kidney disease and/or diabetes have a risk of almost 50%. Currently, there is no effective medication used to treat CI-AKI, which emphasizes the need for clinical efforts to prevent its development prior to diagnosis. The inflammation response has been found to significantly impact the development of CI-AKI.WBC count, leukocyte count, platelet-to-lymphocyte ratio, and WBC differential are widely recognized as indications of inflammation. |
| Objectives | 3 | State specific objectives, including any prespecified hypotheses | 3 | this study aimed to investigate the relationships between pre-procedure WBC count and CI-AKI incidence in patients with coronary artery disease receiving PCI. |
| Methods | | | |  |
| Study design | 4 | Present key elements of study design early in the paper | 3 | This study was a prospective observation study of consecutive patients who underwent PCI at 2 clinical centers in China |
| Setting | 5 | Describe the setting, locations, and relevant dates, including periods of recruitment, exposure, follow-up, and data collection | 3-5 | A prospective observation study of consecutive patients who underwent PCI at 2 clinical centers in China between September 1, 2015, and September 1, 2017. Adult patients (18 years of age or older) with documented serum creatinine (SCr) records, both pre- and post-PCI (within 72 hr after the procedure), were included.  CI-AKI was defined as an increase in serum creatinine by ≥ 0.5mg/dl or a relative ≥ 25% within 72 hours following the procedure, without another clear cause for acute kidney injury. All patients underwent a follow-up evaluation at the 1-year mark, either during a clinical visit or via telephone contact. An independent clinical events committee, unaware of the details of the study, evaluated all adverse events. |
| Participants | 6 | (*a*) *Cohort study*—Give the eligibility criteria, and the sources and methods of selection of participants. Describe methods of follow-up  *Case-control study*—Give the eligibility criteria, and the sources and methods of case ascertainment and control selection. Give the rationale for the choice of cases and controls  *Cross-sectional study*—Give the eligibility criteria, and the sources and methods of selection of participants | 3-5 | Adult patients (18 years of age or older) with documented serum creatinine (SCr) records, both pre- and post-PCI (within 72 hr after the procedure), were included. The exclusion criteria for the study included individuals who had failed PCI, those with hemodynamic instability or class IV heart failure(as defined by the NYHA functional classification system), stage 4 or higher chronic kidney disease, hypersensitivity to contrast medium, anyone who had been exposed to a second contrast medium within one week of the exposure to the first, and those who had received metformin or aminophylline within the 2 weeks prior to PCI.  All patients who were treated with drug-eluting stents and received dual antiplatelet therapy (aspirin associated with clopidogrel or ticagrelor) were recommended for 12 months. At the time of hospital admission, patients received a loading dose of 300mg aspirin followed by 100mg/day and either a loading dose of 300mg clopidogrel followed by 75mg/day or a loading dose of 180mg ticagrelor followed by 90mg/day as P2Y12 inhibitor. PCI was performed according to standard clinical practice, and PCI procedures were achieved through the radial approach by experienced interventional cardiologists. Professional operators and radiologists can choose to use Visipaque or Iohexol for contrast-enhanced procedures. Procedural anticoagulation was performed by administering unfractionated heparin to target an activated clotting time of 250 to 300 s. At the decision of the professional operator’s discretion, perioperative statin (rosuvastatin 20mg/day or atorvastatin 10mg/day), ACEI/ARB inhibitor, beta-blocker, proton pump inhibitor, and intra coronary or intravenous glycoprotein IIb/IIIa inhibitor was also administered during PCI.  All patients were given intravenous hydration. Standard hydration therapy, using saline or bicarbonate solution, was administered at the physician's discretion. All patients received pre- and post-procedural hydration with saline or bicarbonate solution at a rate of 1mL/kg/h for 12 hours. If a patient’s ejection fraction (EF) was below 40% during PCI, hydration was initiated at a rate of 0.5 mL/kg/hr and continued for 18 to 24 hours post-emergency PCI.  All patients underwent a follow-up evaluation at the 1-year mark, either during a clinical visit or via telephone contact. An independent clinical events committee, unaware of the details of the study, evaluated all adverse events. |
|  |  | (*b*) *Cohort study*—For matched studies, give matching criteria and number of exposed and unexposed  *Case-control study*—For matched studies, give matching criteria and the number of controls per case |  |  |
| Variables | 7 | Clearly define all outcomes, exposures, predictors, potential confounders, and effect modifiers. Give diagnostic criteria, if applicable | 4 | CI-AKI was defined as an increase in serum creatinine by ≥ 0.5mg/dl or a relative ≥ 25% within 72 hours following the procedure, without another clear cause for acute kidney injury. |
| Data sources/ measurement | 8* | For each variable of interest, give sources of data and details of methods of assessment (measurement). Describe comparability of assessment methods if there is more than one group | 4-5 | Blood samples were collected at admission and 48-72 hours after CM administration to measure Scr concentrations. To define CI-AKI, the highest Scr value after the procedure was considered. Upon hospital admission, a specific clinical laboratory conducted a baseline measurement of the patient's WBC count. Upon hospital admission, a specific clinical laboratory conducted a baseline measurement of the patient's WBC count. in the Third Xiangya Hospital of Central South University. Serum glucose levels and lipid profiles were measured while patients were fasting in the morning after administration. The estimated Glomerular Filtration Rate(eGFR )was calculated using the Modification of diet in renal disease study equation(MDRD). All patients underwent a follow-up evaluation at the 1-year mark, either during a clinical visit or via telephone contact. An independent clinical events committee, unaware of the details of the study, evaluated all adverse events. |
| Bias | 9 | Describe any efforts to address potential sources of bias | 5 | Upon hospital admission, a specific clinical laboratory conducted a baseline measurement of the patient's WBC count. Upon hospital admission, a specific clinical laboratory conducted a baseline measurement of the patient's WBC count. in the Third Xiangya Hospital of Central South University. Serum glucose levels and lipid profiles were measured while patients were fasting in the morning after administration. An independent clinical events committee, unaware of the details of the study, evaluated all adverse events. |
| Study size | 10 | Explain how the study size was arrived at | 5-6 | We predict a sensitivity of 90% and specificity of 90%, while the prevalence rate of CI-AKI was 5%. the required sample size was finally calculated to be 728. The percentage of missing values was less than 1% for all variables in the study. |

Continued on next page

| Quantitative variables | 11 | Explain how quantitative variables were handled in the analyses. If applicable, describe which groupings were chosen and why | 5 | Continuous variables were summarized by means ± SD and median (interquartile range). The normality of continuous data was assessed using the Shapiro-Wilks test and the Kolmogorov-Smirnov test. Continuous variables normally distributed were compared using an unpaired student’t-test, while non-normal variables were compared using the Mann-Whitney U-test. |
| --- | --- | --- | --- | --- |
| Statistical methods | 12 | (*a*) Describe all statistical methods, including those used to control for confounding | 5 | SPSS 25.0 was used to perform statistical calculations. |
|  |  | (*b*) Describe any methods used to examine subgroups and interactions | 5 | The association between categorical variables and treatment groups was analyzed using Chi-squared or Fisher exact tests. In determining the factors independently associated with CI-AKI, we considered a comprehensive list of patient characteristics and treatment patterns as candidates. Using forward elimination, a multivariate logistic regression model in which all variables were associated with CI-AKI was developed. During the multivariable model-building process, variables with a P value ≤ 0.15 and those considered clinically important, biologically plausible, or supported by previously published data were included.Using Variance Inflation Factors (VIF) to detect multicollinearity. |
|  |  | (*c*) Explain how missing data were addressed | 6 | Missing values of categorical variables were imputed to their most common value, and continuous variables to the median of the non-missing values. |
|  |  | (*d*) *Cohort study*—If applicable, explain how loss to follow-up was addressed  *Case-control study*—If applicable, explain how matching of cases and controls was addressed  *Cross-sectional study*—If applicable, describe analytical methods taking account of sampling strategy | 6 | All patients completed a follow-up evaluation at the one-year mark, either in person or via phone. |
|  |  | (*e*) Describe any sensitivity analyses | 5 | Using forward elimination, a multivariate logistic regression model in which all variables were associated with CI-AKI was developed. During the multivariable model-building process, variables with a P value ≤ 0.15 and those considered clinically important, biologically plausible, or supported by previously published data were included.Using Variance Inflation Factors (VIF) to detect multicollinearity. The adjusted odds ratios were calculated for each of these variables. ROC curve analysis was utilized to assess the ability of baseline WBC count to differentiate between patients with and without CI-AKI. The optimal cut-off point was determined by identifying the baseline WBC count that maximized the sum of specificity and sensitivity. Kaplan-Meier survival charts were created to determine the difference in 1-year death rate between patients with CI-AKI and those without. To compare survival curves, we used the log-rank test. For all statistical tests carried out, two-tailed P values were used, with statistical significance being defined by a P value of ≤ 0.05. |
| Results | | | | |
| Participants | 13* | (a) Report numbers of individuals at each stage of study—eg numbers potentially eligible, examined for eligibility, confirmed eligible, included in the study, completing follow-up, and analysed | 6 | Two centers enrolled 1300 patients for PCI between September 2015 and July 2017. Of these, 1013 patients met the study criteria(Fig.1.). All patients completed a follow-up evaluation at the one-year mark, either in person or via phone. |
|  |  | (b) Give reasons for non-participation at each stage | Figure1 | 158 opatients not meeting inclusion criteria;87patients meeting exclusion criteria;42 patients declined to participate |
|  |  | (c) Consider use of a flow diagram | Figure1 |  |
| Descriptive data | 14* | (a) Give characteristics of study participants (eg demographic, clinical, social) and information on exposures and potential confounders | 6 | the baseline clinical characteristics stratified by CI-AKI, including patient demographics, clinical, biochemical, angiographic, and procedural variables. |
|  |  | (b) Indicate number of participants with missing data for each variable of interest | 6 | The percentage of missing values was less than 1% for all variables in the study. Missing values of categorical variables were imputed to their most common value, and continuous variables to the median of the non-missing values. |
|  |  | (c) *Cohort study*—Summarise follow-up time (eg, average and total amount) | 6 | All patients completed a follow-up evaluation at the 1-year mark |
| Outcome data | 15* | Cohort study—Report numbers of outcome events or summary measures over time | 6 | Patients with CI-AKI (referred to as the “CI-AKI group” hereafter) had a significantly higher WBC count than those without CI-AKI (referred to as the “Non-CI-AKI group” hereafter) (10.41 ± 5.37 vs. 8.09± 3.10 103mL, respectively; P= 0.004) and had a significantly lower chance of having used statins (P= 0.022) compared with patients in the Non-CI-AKI group. |
|  |  | *Case-control study—*Report numbers in each exposure category, or summary measures of exposure |  |  |
|  |  | *Cross-sectional study—*Report numbers of outcome events or summary measures |  |  |
| Main results | 16 | (*a*) Give unadjusted estimates and, if applicable, confounder-adjusted estimates and their precision (eg, 95% confidence interval). Make clear which confounders were adjusted for and why they were included | 6-7 | Female patients, low blood pressure, peripheral vascular disease (PVD), intra-aortic balloon pump (IABP), heart failure (I-III), lower hemoglobin, and higher fasting glucose were more likely to develop CI-AKI. |
|  |  | (*b*) Report category boundaries when continuous variables were categorized | 6-7 | ROC curve analysis of pre-procedural WBC count found that pre-procedural WBC count could be used to predict CI-AKI incidence. [area under the curve = 0.684 (95% confidence interval 0.545-0.723), P= 0.002] (Fig.2.). A cut-off value of ≥ 11.03×10^9/L was identified as optimal in predicting CI-AKI incidence, providing a sensitivity of 41.0% and specificity of 86.5%. |
|  |  | (*c*) If relevant, consider translating estimates of relative risk into absolute risk for a meaningful time period | 6-7 | Apart from low Hb (OR 0.97,95% CI 0.95-0.99; P=0.002), fasting glucose (OR 1.10,95% CI 1.00-1.20; P=0.043), Iohexol (OR 4.55,95% CI 1.37-15.14; P=0.014), WBC count (OR 1.12,95% CI 1.03-1.21; P=0.006) remained significant and independent in predicting the occurrence of CI-AKI. The use of statins was identified as effective in preventing CI-AKI development (OR 0.24,95% CI 0.05-1.21; P=0.044). |

Continued on next page

| Other analyses | 17 | Report other analyses done—eg analyses of subgroups and interactions, and sensitivity analyses | NA |  |
| --- | --- | --- | --- | --- |
| Discussion | | | | |
| Key results | 18 | Summarise key results with reference to study objectives | 7 | A cut-off value of ≥ 11.03×10^9/L was identified as optimal in predicting CI-AKI incidence, providing a sensitivity of 41.0% and specificity of 86.5%. |
| Limitations | 19 | Discuss limitations of the study, taking into account sources of potential bias or imprecision. Discuss both direction and magnitude of any potential bias | 9-10 | This study is not without limitations. First, it is important to note, however, that this study was merely observational and was conducted in two medical centers with a small sample size which may have affected our results; Second, we excluded patients without measured plasma creatine upon admission or 24-72h after PCI procedure, which may introduce selection bias; Third, C-reactive protein (CRP) is the important of inflammatory factor, However, we just test CRP levels in some high-risk patients. This study would be more convincing with a detailed analysis of cytokines, for example, TNF-α, IL1, and IL6 in the plasma. However, this is just an observation study, so we didn’t test TNF-α, IL1, and IL6 in patients; Finally, Patients who developed CI-AKI showed significant differences in their baseline as compared to those who did not. Despite controlling for potential confounding factors related to known comorbidities and drug use, we cannot completely eliminate the possibility of unmeasured confounders affecting our results. Therefore, our findings should be considered as a hypothesis for further confirmation through additional studies. |
| Interpretation | 20 | Give a cautious overall interpretation of results considering objectives, limitations, multiplicity of analyses, results from similar studies, and other relevant evidence | 9 | WBC count is an independent and powerful predictor of risk for CI-AKI in patients undergoing PCI. ROC curve analysis showed that a baseline WBC count of 11.03 was a fair preliminary indicator of creatinine increases, and patients who experienced CI-AKI had a notably lower 1-year survival rate compared to those who did not suffer from CI-AKI. |
| Generalisability | 21 | Discuss the generalisability (external validity) of the study results | 9 | There is mounting evidence supporting the use of WBC count as a prognostic marker for determining CI-AKI risk in patients who receive PCI. To effectively use this marker in clinical settings, further studies are needed to establish treatment options, identify anti-inflammatory medications, and compare its utility with other known inflammatory markers. |
| Other information | |  | | |
| Funding | 22 | Give the source of funding and the role of the funders for the present study and, if applicable, for the original study on which the present article is based | 10 | This work was supported by the National Natural Science Foundation of China (No. 81803639) |

*Give information separately for cases and controls in case-control studies and, if applicable, for exposed and unexposed groups in cohort and cross-sectional studies.

**Note:** An Explanation and Elaboration article discusses each checklist item and gives methodological background and published examples of transparent reporting. The STROBE checklist is best used in conjunction with this article (freely available on the Web sites of PLoS Medicine at http://www.plosmedicine.org/, Annals of Internal Medicine at http://www.annals.org/, and Epidemiology at http://www.epidem.com/). Information on the STROBE Initiative is available at www.strobe-statement.org.
