# Supplementary material for: Impact of white blood cell count on the development of contrast-induced acute kidney injury in patients receiving percutaneous coronary intervention
Source: PeerJ. 2024 Jun 28;12:e17493. doi: 10.7717/peerj.17493 (PMC11636986; doi:10.7717/peerj.17493)
Supplement: Supplemental Information 3 [file peerj-12-17493-s003.pdf]

行 PCI 术的冠心病患者登记研究知情同意书

姓 名： 性 别： 年 龄：

住院号： 临床诊断： 联系方式：

第一联系人： 联系方式：

地址：_______省_______市_______区（镇）__________________________________

尊敬的病友及家属：

抗血小板治疗是冠心病预防和治疗的基石。对于接受经皮冠状动脉介入治疗（PCI）的冠心病患者，目前指南常规推荐双重抗血小板治疗，即小剂量阿司匹林与噻吩并吡啶类药物（如氯吡格雷）联用使用。为了解您用药后血小板的功能情况，更科学、合理地应用抗血小板药物，我院开展了行 PCI 术的冠心病患者登记研究。

该研究为临床观察性登记研究。参与该研究并无任何后果。研究中使用的任何药物均是您的主治医生结合您的病情给予的最适治疗方案，我们仅收集您在治疗过程中的相关临床数据，以及您的与本次治疗有关的基因信息。如您参加本研究，您将获得免费的术后一个月血小板功能检测。

如果您对上述情况表示理解，愿意参加该项研究，请在指定处签名。谢谢！

患者签字： 日期： 年 月 日

如果患者无法进行知情同意，则由法定代理人代理进行

法定代理人签字： 与患者关系：

日期： 年 月 日

患者联系方式：

我已向该受试者充分解释和说明了本临床研究的目的、操作过程以及受试者参加该实验可能存在的相关问题和潜在利益，并满意的回答了受试者所有相关问题。

医生签字： 日期： 年 月 日

医生联系方式：
